# Pyrazolate-Bridged PdCo and PdCu Complexes as Dual DNA-Binding and ROS-Driven Cytotoxic Agents

**DOI:** 10.3390/ijms27209009

**Published:** 2026-10-11

**Authors:** Marina A. Uvarova, Vitalia V. Kulikova, Yaroslav V. Kozmenko, Svetlana V. Revtovich, Arina D. Filippova, Maxim A. Shmelev, Darina V. Sokolova, Vadim S. Pokrovsky, Alexandra P. Kaverina, Pavel N. Solyev, Igor L. Eremenko

**Affiliations:** 1 Kurnakov Institute of General and Inorganic Chemistry, Russian Academy of Sciences, Leninsky Prosp. 31, GSP-1, 119991 Moscow, Russiashmelevma@yandex.ru (M.A.S.); ilerem@igic.ras.ru (I.L.E.); 2 Engelhardt Institute of Molecular Biology, Russian Academy of Sciences, Vavilova Str., 32, 119991 Moscow, Russia; vitviku@yandex.ru (V.V.K.); kozmenko@eimb.ru (Y.V.K.); kaverina.a@list.ru (A.P.K.); solyev@gmail.com (P.N.S.); 3 N.N. Blokhin National Medical Research Center of Oncology, Kashirskoe Shosse 23, 115522 Moscow, Russia; d.v.sokolova@gmail.com (D.V.S.); vadimpokrovskky@gmail.com (V.S.P.); 4 Peoples’ Friendship University of Russia, Miklukho-Maklaya 6, 117198 Moscow, Russia

**Keywords:** heterometallic compounds, palladium(II), crystal structure, anticancer activity, ROS, docking

## Abstract

Heterometallic complexes offer a promising anticancer strategy owing to their simultaneous action on multiple biological targets. In this work, we report the synthesis and evaluation of the antitumor potential of two heterometallic pyrazolate-bridged complexes, [PdCo(phen)(µ-dmpz)_2_(OOCMe)_2_] (**PdCo**) and [PdCu(phen)(µ-dmpz)_2_(OOCMe)_2_] (**PdCu**) (phen = 1,10-phenanthroline, dmpz = 3,5-dimethylpyrazole). The complexes were comprehensively characterized using a combined spectroscopic and analytical approach, including single-crystal X-ray diffraction (SC-XRD), PXRD, FTIR, UV-Vis, and mass spectrometry, confirming their structural integrity in both solid state and solution. Their cytotoxicity was assessed in vitro against colon, A549 lung, and breast cancer cell lines, as well as normal human dermal fibroblasts. The complexes exhibited micromolar IC_50_ values with selectivity indices up to 7. Using circular dichroism (CD) and fluorescence spectroscopy, it was demonstrated that **PdCo** binds to DNA, with minor groove binding (K_d_ = 7.6 μM) being the preferred mode of interaction, dominating over intercalation (K_d_ = 26.1 μM). Molecular docking simulations confirmed that the dissociated [Pd(phen)(µ-dmpz)_2_] fragment is capable of binding to DNA. Furthermore, chemiluminescence assays revealed pronounced peroxidase-like and superoxide dismutase-like activities of **PdCu** and **PdCo**, implicating ROS-mediated oxidative stress as a second, complementary cytotoxic pathway. Collectively, these findings highlight the dual-action mechanism of the synthesized complexes, combining DNA targeting with ROS-mediated oxidative damage, positioning them as highly potent candidates for further anticancer development.

## 1. Introduction

In recent years, heterometallic complexes have emerged as a promising strategy in anticancer research [1]. Their unique advantage lies in their ability to act on multiple biological targets simultaneously, which can potentially circumvent resistance mechanisms and enhance selectivity toward cancer cells [2]. By incorporating two different metal centers within a single molecular scaffold, such complexes can induce synergistic effects—for example, one metal center may interact with DNA while the other promotes the generation of reactive oxygen species (ROS), leading to combined genotoxic and oxidative stress in cancer cells [3,4,5].

Pyrazolate-bridged heterometallic complexes are particularly attractive due to their structural stability, tunable electronic properties, and ability to maintain close proximity between two distinct metal ions [6,7,8,9,10]. In this context, palladium(II) complexes have gained attention as DNA-binding agents, often showing faster ligand exchange kinetics compared to platinum(IV) analogs [11,12,13,14,15]. Meanwhile, 3d transition metals such as cobalt and copper are well-known for their redox activity and capacity to catalyze the formation of ROS, including hydroxyl radicals and superoxide anions, thereby inducing oxidative damage within malignant cells [16,17,18,19,20,21]. It has been well established that copper, cobalt, and palladium complexes bearing pyrazole moieties possess antiproliferative as well as antibacterial properties [22,23,24,25,26,27,28,29].

Despite these promising features, the biological activity of heterometallic pyrazolate-bridged complexes has remained virtually unexplored [29,30]. This knowledge gap is particularly significant given that the combination of a palladium fragment (capable of DNA interaction) with a redox-active 3d metal center (capable of ROS generation) within a single, stable pyrazolate-bridged scaffold could provide a synergistic dual-action mechanism against cancer cells. Thus, the present work represents the first systematic investigation of the bioactivity of heterometallic pyrazolate-bridged complexes. Therefore, the aim of this work was to obtain heterometallic pyrazolate-bridged complexes that, thanks to the combination of active metal fragments, would be capable of exerting a dual effect on cells.

Herein, we report synthesis and characterization of the novel heterometallic pyrazolate-bridged complex [PdCo(phen)(µ-dmpz)_2_(OOCMe)_2_] (**PdCo**), similar in structure to the recently described complex [PdCu(phen)(µ-dmpz)_2_(OOCMe)_2_] (**PdCu**) [6]. The complex was characterized by single-crystal X-ray diffraction (SC-XRD), IR spectroscopy, and mass spectrometry. The cytotoxicity of **PdCo** and **PdCu** was evaluated in vitro against three cancer cell lines—HCT116 (colon), A549 (lung), and SKBR3 (breast)—as well as in normal human dermal fibroblasts (HDFs). Both complexes demonstrated micromolar IC_50_ values. To elucidate their mechanism of action, we investigated DNA binding via molecular docking simulations, which suggest that the dissociated [Pd(phen)(µ-dmpz)_2_] fragment is capable of binding to DNA. Furthermore, chemiluminescence assays revealed pronounced peroxidase-like and superoxide dismutase-like activities of complexes **PdCo** and **PdCu**, implicating ROS-mediated oxidative stress as a second, complementary cytotoxic pathway. Together, these findings provide the first experimental evidence for the anticancer potential of heterometallic pyrazolate-bridged complexes and position them as promising dual-action anticancer candidates worthy of further preclinical development.

## 2. Results and Discussion

### 2.1. Synthesis and Characterization of Compounds

The synthesis of the heterometallic complexes was accomplished using a methodology previously described by our group [6]. This approach is based on the deprotonation of a pyrazole ligand initially coordinated to a first-row transition metal center, which is promoted by a Lewis-acidic Pd(II) fragment. The coordination environment of the palladium center is partially blocked by a rigid bidentate N-donor ligand, an arrangement that curtails uncontrolled oligomerization and facilitates the isolation of discrete binuclear complexes.

The reactions of [Pd(phen)(OOCMe)_2_] with the copper precursor [Cu(Hdmpz)_2_(OOCMe)_2_] afford the binuclear complex [PdCu(phen)(μ-dmpz)_2_(OOCMe)_2_] (**PdCu**). The crystal structure and characterization of the **PdCu** complex have been reported previously by our group [6].

**Scheme 1:**
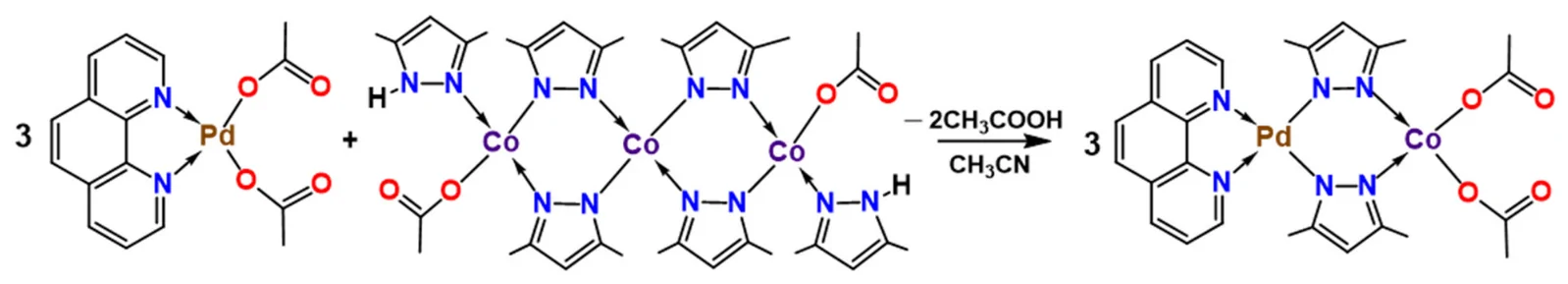
Synthesis of **PdCo**.

The interaction of cobalt(II) complex [Co_3_(μ-dmpz)_4_(Hdmpz)_2_(OOCMe)_2_] [31] with [PhenPd(OOCMe)_2_] leads to similar binuclear complexes [Pd(phen)Co(μ-dmpz)_2_(OOCMe)_2_] (**PdCo**) (Scheme 1).

**Figure 1:**
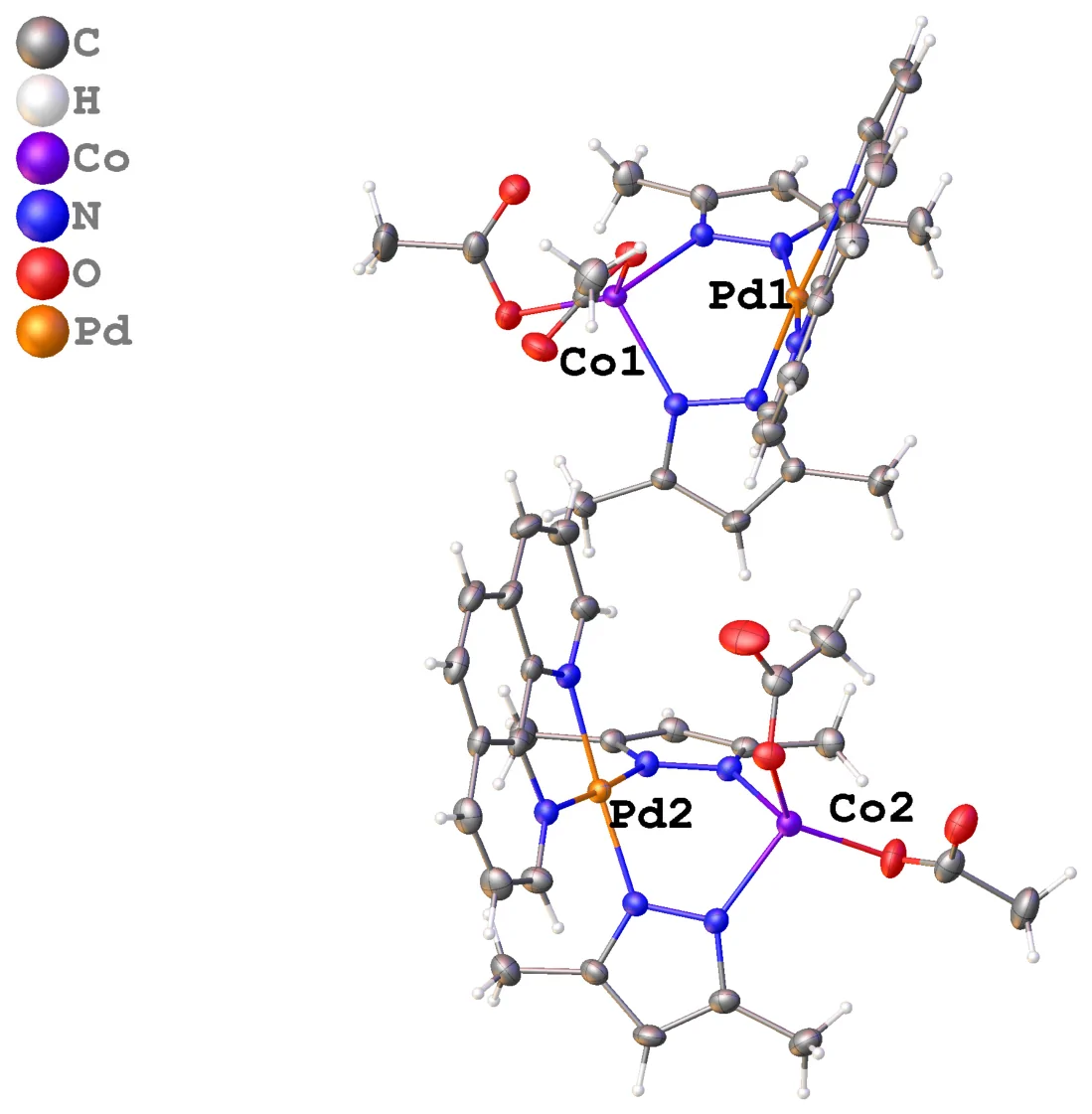
The molecular structure of **PdCo** complex with thermal ellipsoids drawn at the 50% probability level.

According to X-ray diffraction data, the unit cell of the **PdCo** complex contains two crystallographically independent molecules, each representing a binuclear pyrazolate-bridged species. The structure of **PdCo** (Figure 1) features the bis(pyrazolate)palladium fragment chelated by phenanthroline and bridged to a cobalt(II) center. Palladium retains its square-planar geometry, with Pd–N distances (1.977(4)–2.063(4) Å) closely matching those observed in the copper analogues (Table S1) [6]. The cobalt center is also composed of four coordinates—two nitrogen atoms from pyrazolate ligands and two oxygen atoms from acetate groups forming a tetrahedral environment, with Co–N and Co–O bond lengths typical of high-spin Co(II). The Pd···Co distance of 3.215(4) Å confirms the occurrence of a non-bonding metal–metal interaction.

The crystal packing of the synthesized compound is stabilized by a system of π···π interactions between pairs of phen molecules (Table S3) and a system of C–H···O interactions (Table S4) between hydrogen atoms of the phen molecules and oxygen atoms of the carboxyl groups; collectively, these interactions stabilize a supramolecular chain structure (Figure S1).

In the IR spectrum of **PdCo**, bands are observed at 3429 cm^−1^ (w, ν(O–H) of residual water/acetic acid), 3053 cm^−1^ (w, ν(C–H) of aromatic rings), and 2918 cm^−1^ (w, ν(C–H) of methyl groups of acetate). The most intense bands in the 1623–1389 cm^−1^ region (1623 s, 1576 s, 1521 s, 1415 s, 1389 s) are assigned to ν(C=N)/ν(C=C) stretching vibrations of pyridine and pyrazolate rings, as well as to asymmetric and symmetric ν(COO^−^) modes of acetate groups (Figure S2).

The phase purity of the sample **PdCo** was confirmed by powder X-ray diffraction analysis (Figure S5).

The composition of the complex in solution was confirmed by mass spectrometry in acetonitrile. The ESI-MS spectrum of **PdCo** displays characteristic ions at *m/z* 382 [PhenPd(dmpz)]^+^, *m/z* 477 supports the presence of the [Pd(phen)(dmpz)_2_]^+^ and signals assigned to [PdCo(phen)(μ-dmpz)_2_(OOCMe)]^+^ (*m/z* 593.8) (Figure S3). It can be assumed that the weak signals at *m/z* 807 correspond to the associate {[PdCo(phen)(μ-dmpz)_2_(OOCMe)_2_](Hdmpz)(CH_3_COOH)}^+^, where CH_3_COOH is acetic acid formed during the reaction.

**Figure 2:**
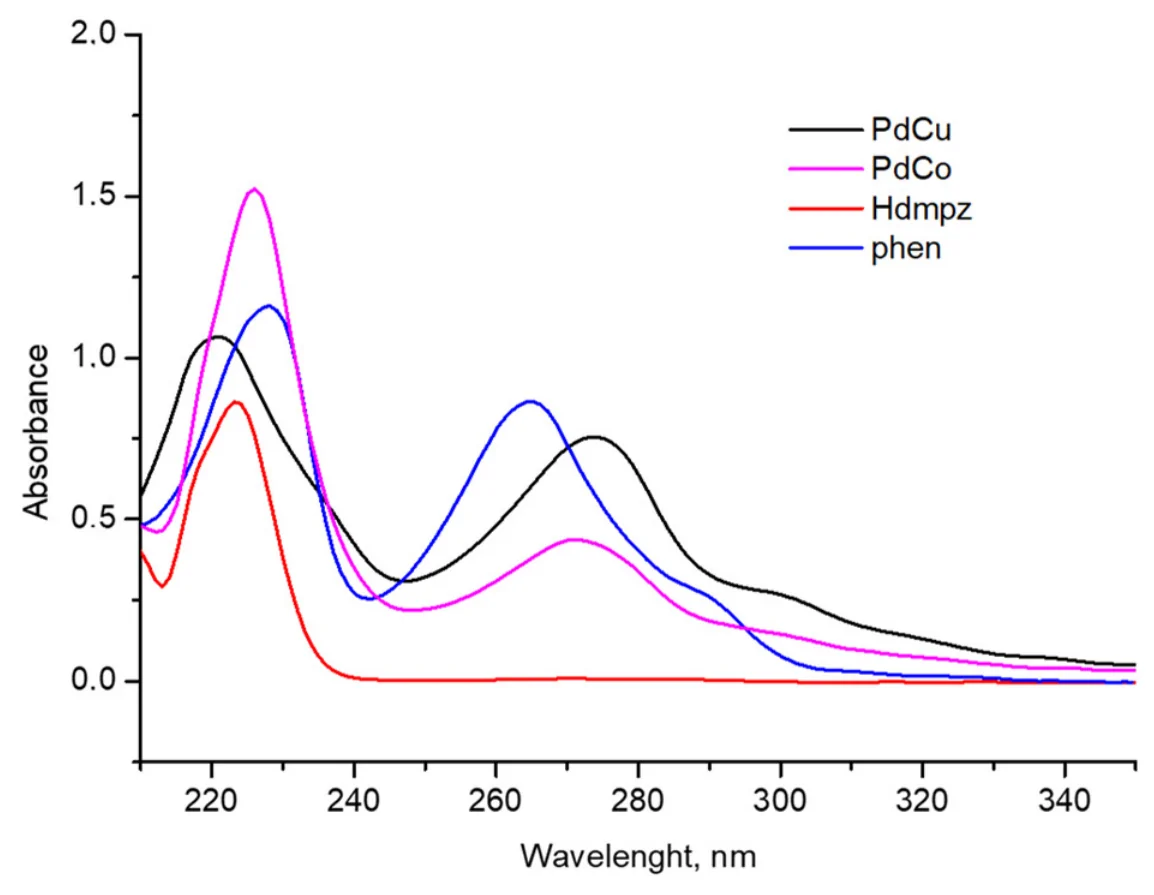
UV-vis spectra of **PdCu**, **PdCo** and free ligands phen and Hdmpz (C = 10^−5^ M) in 1% DMSO/H_2_O solution at room temperature.

To confirm the retention of the coordination environment of Pd^II^ in solution, the electronic absorption spectra of **PdCo**, **PdCu**, and the free ligands phen and Hdmpz were recorded in 1% DMSO/H_2_O at room temperature (c = 10^−5^ M) (Figure 2).

The free phenanthroline displays characteristic π→π* transitions at ~230 and 265 nm, while Hdmpz shows a single intense peak at ~225 nm. Upon complexation, the π→π* bands of phen undergo a clear bathochromic shift to ~272 nm in both **PdCo** and PdCu. This red shift arises from dπ(Pd)→π*(phen) back-donation, which stabilizes the chelate ring and unequivocally confirms that phen remains firmly coordinated to the Pd(II) center in the aqueous DMSO medium. Importantly, the shift in absorption bands characteristic of the free ligands rules out any significant ligand dissociation, indicating the stability of the coordination sphere in solution. UV-vis spectra of **PdCo** in comparison with the starting compounds [Pd(phen)(OAc)_2_] and [Co_3_(μ-dmpz)_4_(Hdmpz)_2_(OAc)_2_] are also presented in Figure S6.

These spectroscopic findings are fully consistent with the mass-spectrometric data, which unambiguously confirm the presence of the [Pd(phen)(dmpz)_2_] fragment as the core structural unit. The combination of MS and UV-Vis thus provides compelling evidence that the coordination of both phenanthroline and pyrazolate ligands to palladium is fully preserved in the 1% DMSO/H_2_O solution.

### 2.2. Cytotoxicity

The cytotoxicity of two novel heterobimetallic complexes, **PdCo** and **PdCu**, and starting compounds [Pd(phen)(OAc)_2_] and [Co_3_(μ-dmpz)_4_(Hdmpz)_2_(OAc)_2_] was evaluated against three human cancer cell lines (HCT116 (colon), A549 (lung), SKBR3 (breast) and normal human fibroblasts (HDFs) in comparison with cisplatin (Table 1, Figure S4).

The greatest activity was demonstrated by the **PdCu** complex. Its IC_50_ values were 3.01 µM for HCT116 and 2.31 µM for A549—approximately 2.5 and 2.2-fold lower than those of cisplatin (*p* < 0.05). Moreover, **PdCu** was less toxic to normal cells (IC_50_ = 16.12 µM), resulting in selectivity indices (SIs) for HCT116 and A549 of 5.4 and 7.0, respectively, exceeding those of cisplatin (SI = 3.0 and 5.0, *p* = 0.0037). This indicates a broader therapeutic window for the **PdCu** complex against colorectal and non-small cell lung cancer.

In contrast, the **PdCo** complex showed moderate activity against HCT116 and A549 but exhibited exceptional selectivity toward the SKBR3 breast cancer cell line: IC_50_ = 17.67 µM vs. 136.6 µM in fibroblasts. The resulting selectivity index (SI = 7.7) was 2.7-fold higher than that of cisplatin (SI = 2.9), making PdCo a promising candidate for targeted therapy of HER2-positive breast cancer.

Comparing the cytotoxicity of **PdCo** with the starting compounds reveals clear advantages of the heterometallic complex. The starting [Pd(phen)(OAc)_2_] showed moderate activity against all three cancer cell lines (IC_50_ = 8–12 µM)) with low selectivity (SI = 2.2–3.3), whereas the cobalt precursor was essentially inactive. The **PdCo** complex, in contrast, demonstrated pronounced selectivity toward the SKBR3 cell line (IC_50_ = 18 ± 1 µM), with a selectivity index of 7.7, significantly exceeding both starting compounds. At the same time, its activity against HCT116 and A549 was lower than that of the palladium precursor, indicating a change in the activity profile upon formation of the heterometallic complex. Obviously, the cytotoxicity is not related to a simple addition of the two starting compounds.

Thus, the role of **PdCo** formation is not merely a structural modification but a functional modulation of biological activity. The cobalt center redirects the action of the palladium fragment toward SKBR3 cells, likely through cooperative effects. This results in reduced general toxicity and enhanced selectivity, confirming the rationale for using a heterobimetallic design to create targeted anticancer agents.

The difference in activity between the complexes is likely due to the nature of the partner metal. Copper in **PdCu** may promote the generation of reactive oxygen species and the disruption of copper homeostasis in cancer cells. Cobalt in **PdCo**, on the other hand, forms more stable complexes, reducing overall cytotoxicity but offering high selectivity due to the specific metabolic features of the SKBR3 cell line.

### 2.3. ROS Activity

**Figure 3:**
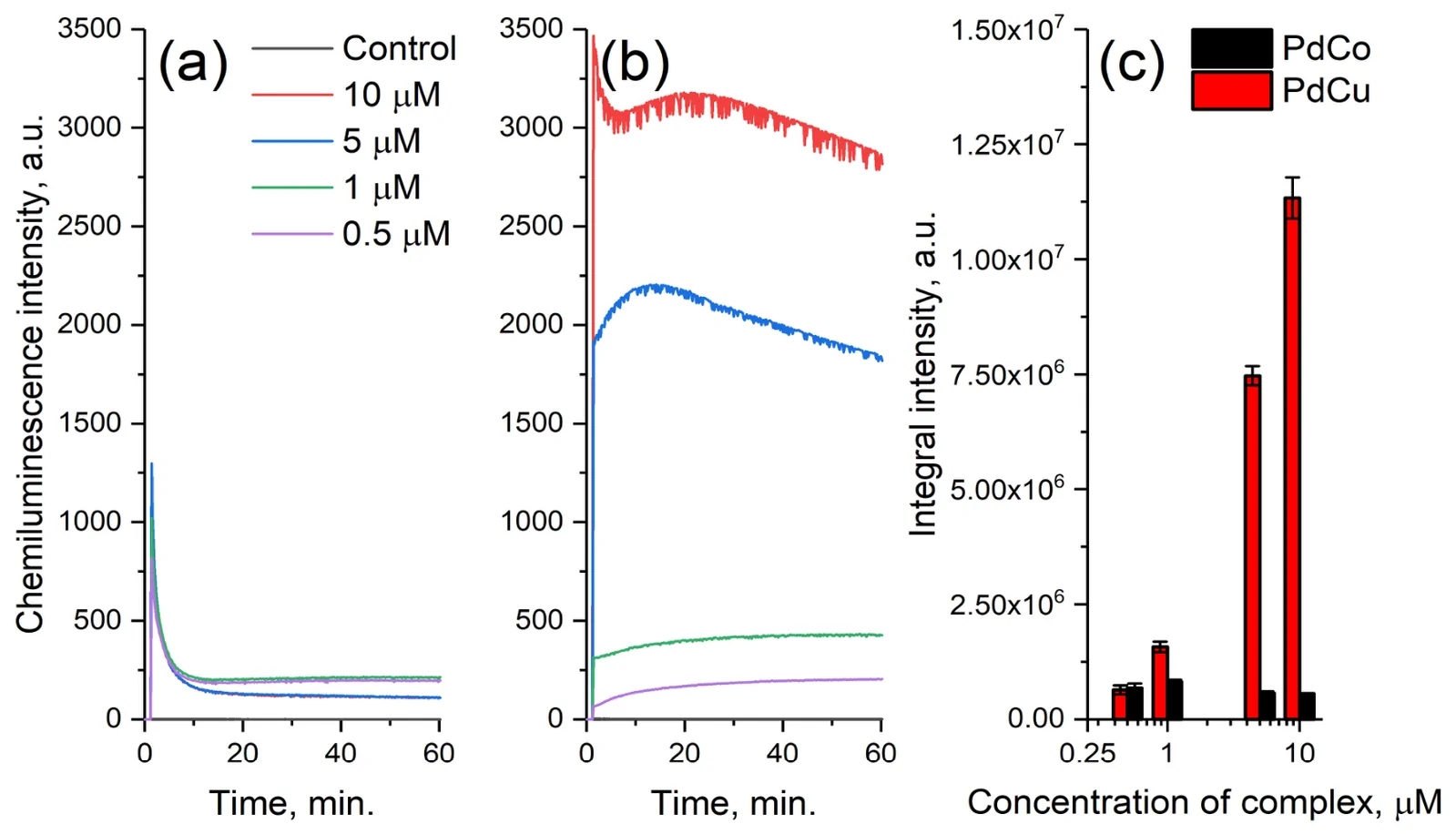
Experimental kinetic curves of luminol chemiluminescence in a phosphate-buffered solution (pH 7.4) containing luminol, hydrogen peroxide and complexes **PdCo** (**a**), **PdCu** (**b**). The intensity of chemiluminescence of luminol on the control curve was no more than 5 a.u. Dependence of the integral chemiluminescence intensity on the complex concentration in reaction mixture (**c**).

Chemiluminescent analysis of radical-scavenging (enzyme-like) activity was performed in a phosphate-buffered solution in two model reactions: oxidation of luminol with hydrogen peroxide (hydroxyl radicals) and oxidation of lucigenin in the presence of xanthine and xanthine oxidase (superoxide anion radicals). From Figure 3, it can be seen that when complex solutions are added to a phosphate buffer containing luminol and hydrogen peroxide, the chemiluminescence intensity increases significantly compared with the control. Thus, the coordination compounds **PdCo** and **PdCu** exhibit pro-oxidant activity with respect to H_2_O_2_. Note that the peroxidase-like activity of the copper complex is two orders of magnitude higher than the activity of the cobalt-containing complex (concentrations of 5 and 10 μM). The pro-oxidant activity of the individual mononuclear palladium complex was found to be almost three orders of magnitude lower than the activity of the heterometallic **PdCo** and **PdCu** complexes (Figure S7). This observation underscores the key role of the heterometal in the pro-oxidant activity of bimetallic complexes. Similarly, Baruah et al. showed that a composite of copper and palladium on a graphene oxide substrate exhibits greater peroxidase-like activity than individual copper and palladium on graphene oxide, and established that hydroxyl radicals formed from hydrogen peroxide under the action of the bimetallic composite play a significant role in peroxidase-like activity [33].

**Figure 4:**
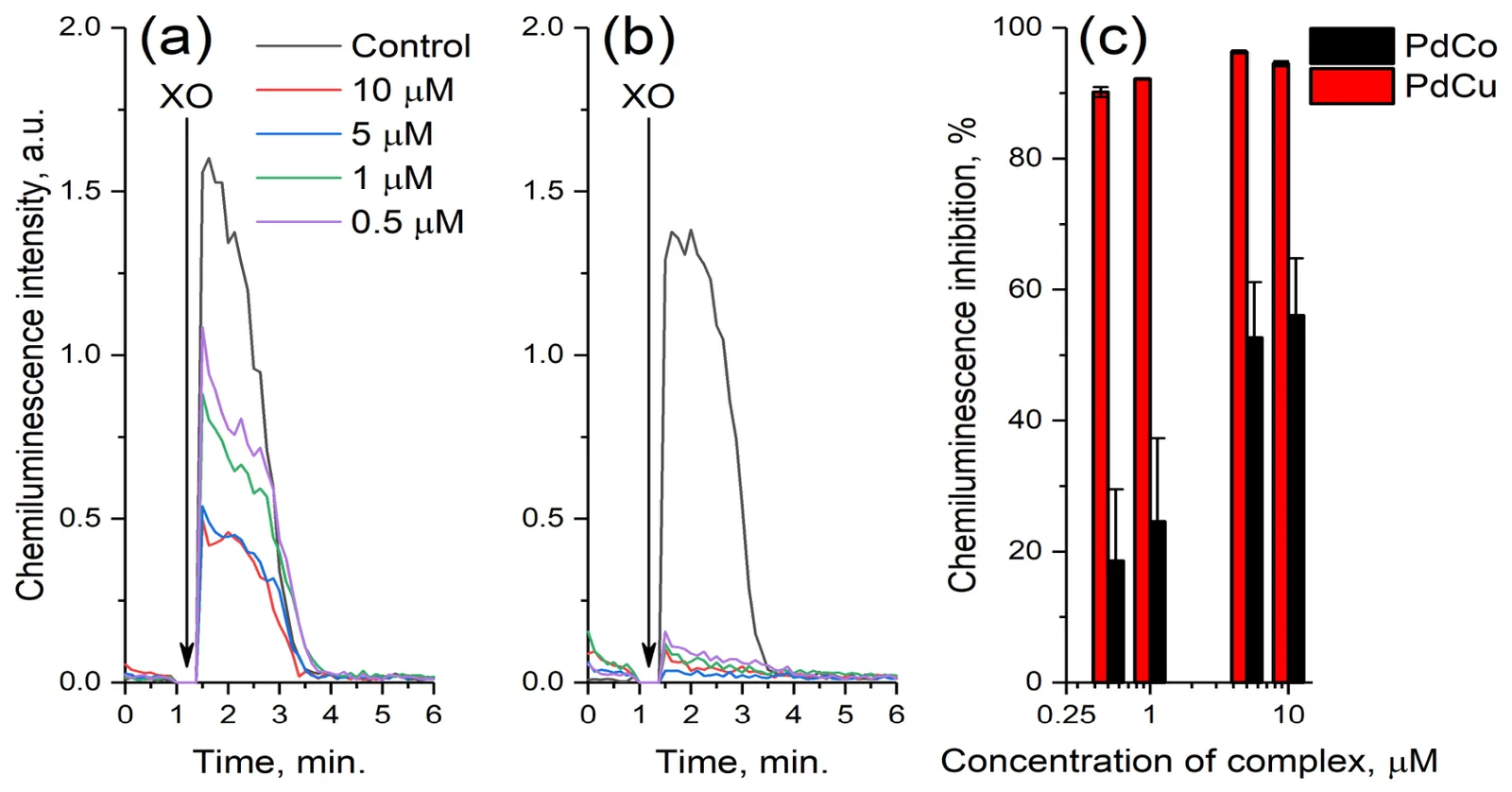
Chemiluminescence curves of lucigenin in phosphate-buffered solution (pH 7.4) in the presence of xanthine, xanthine oxidase (XO), and complexes **PdCo** (**a**), **PdCu** (**b**). Dependence of the degree of chemiluminescence inhibition on the complex concentration in reaction mixture (**c**).

The activity of the complexes with respect to the superoxide anion radical was studied in the model reaction of lucigenin oxidation in the presence of xanthine and xanthine oxidase. As can be seen from Figure 4, the intensity of lucigenin chemiluminescence in the presence of **PdCo** and **PdCu** complexes decreases, which indicates the superoxide dismutase-like activity of the coordination compounds. Notably, without xanthine oxidase, the luminescence intensity of lucigenin is low in the presence of complexes, which indicates the absence of direct oxidative action of **PdCo** and **PdCu** complexes in relation to the chemiluminescent probe. Quantitative analysis showed that the SOD-like activity of the copper-containing complex is more than two times higher than the activity of the cobalt complex (Figure 4c). Copper coordination compounds, including those with pyrazole ligands, are characterized by superoxide dismutase-like activity [34,35,36]. Liu et al. showed that copper doping of platinum nanoparticles increases the SOD-like activity of platinum due to the synergistic effect of the two metals [37]. El-Naggar et al. also found that the superoxide dismutase-like and antitumor activity of copper complexes with 2-methylaminopyridine was higher than the activity of cobalt complexes with a similar ligand [38]. Thus, **PdCu** complex has a more pronounced peroxidase-like and superoxide dismutase-like activity than **PdCo** complex. Both hydroxyl and superoxide anion radicals are present in living systems, and both types of activity can contribute to the overall biological effect of coordination compounds. The complexes can cause cell death both due to the accumulation of hydrogen peroxide by a SOD-like action, and due to the further formation of reactive oxygen species from accumulated H_2_O_2_.

### 2.4. DNA Binding

**Figure 5:**
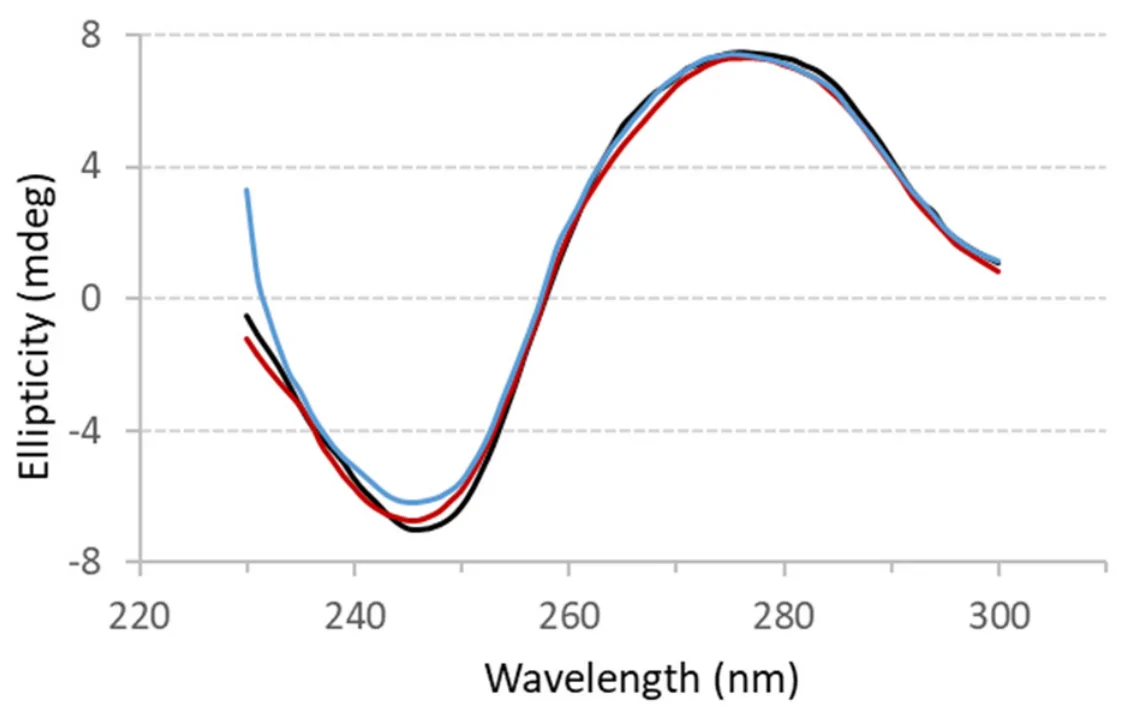
Changes in the CD spectra of salmon sperm DNA (black line) upon the sequential addition of **PdCo** complex in H_2_O. The **PdCo** complex to DNA ratio varied from 1:10 (red line) to 1:5 (blue line).

We used fluorescence spectroscopy and circular dichroism to investigate DNA binding of the **PdCo** complex. To examine the conformational changes in DNA induced by the **PdCo** complex, CD spectra were recorded for salmon sperm DNA over the 230–300 nm range at 25 °C in aqueous solution (Figure 4). The DNA concentration was 50 μM and the **PdCo** complex was added at 5 and 10 μM. The CD spectrum of salmon sperm DNA is in agreement with the canonical B-DNA conformation, as shown in Figure 5. Slight spectral changes around 248 nm may indicate disruptions in the DNA helix and base stacking.

**Figure 6:**
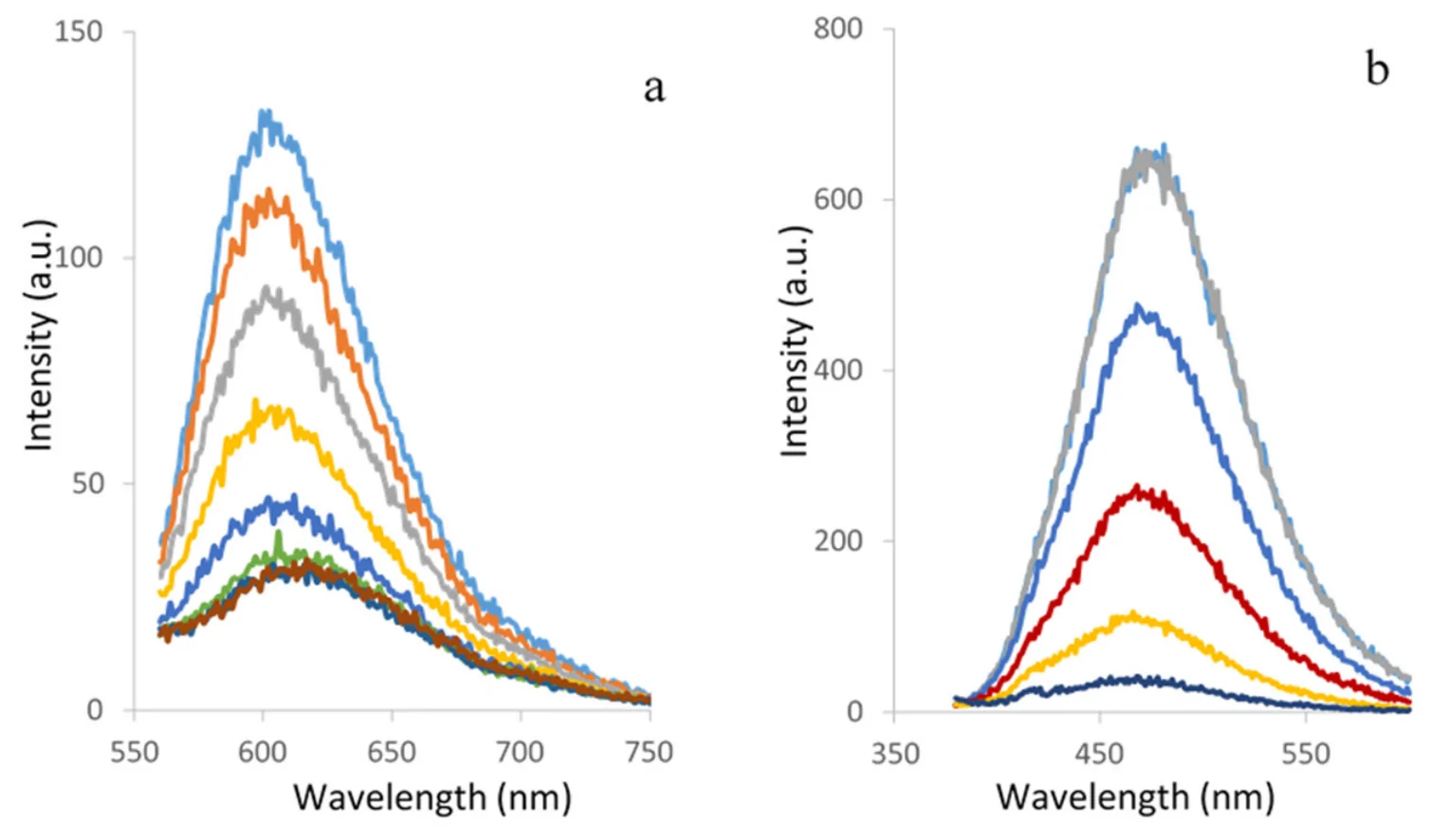
(**a**) Changes in the fluorescent spectra of salmon sperm DNA/ethidium bromide complex upon the sequential addition of the **PdCo** complex. The ratio of **PdCo** complex to DNA was varied from 0:1 to 14:1. (**b**) Changes in the fluorescent spectra of salmon sperm DNA/Hoehcst 33258 complex upon the sequential addition of the **PdCo** complex. The ratio of **PdCo** complex to DNA varied from 0:1 to 20:1.

We investigated whether the **PdCo** complex might enhance the intercalation of the complexes into DNA using fluorescent dye displacement assay with ethidium bromide and Hoechst 33258. This approach is effective for determining the DNA-binding parameters of the compounds lacking their own specific spectral properties that were changed in a different binding environment. Ethidium bromide is a standard dye with an extensively studied intercalation type of binding to DNA, while Hoechst 33258 is a model compound that is used to study ligand interactions in the DNA minor groove [39]. The sequential addition of **PdCo** to salmon sperm DNA with intercalated ethidium bromide resulted in the dose-dependent quenching of the fluorescence at 600 nm (Figure 6a). The addition of **PdCo** to the DNA/Hoechst 33258 complex resulted in the displacement of the dye accompanied by the quenching of fluorescence at 480 nm (Figure 6b).

**Figure 7:**
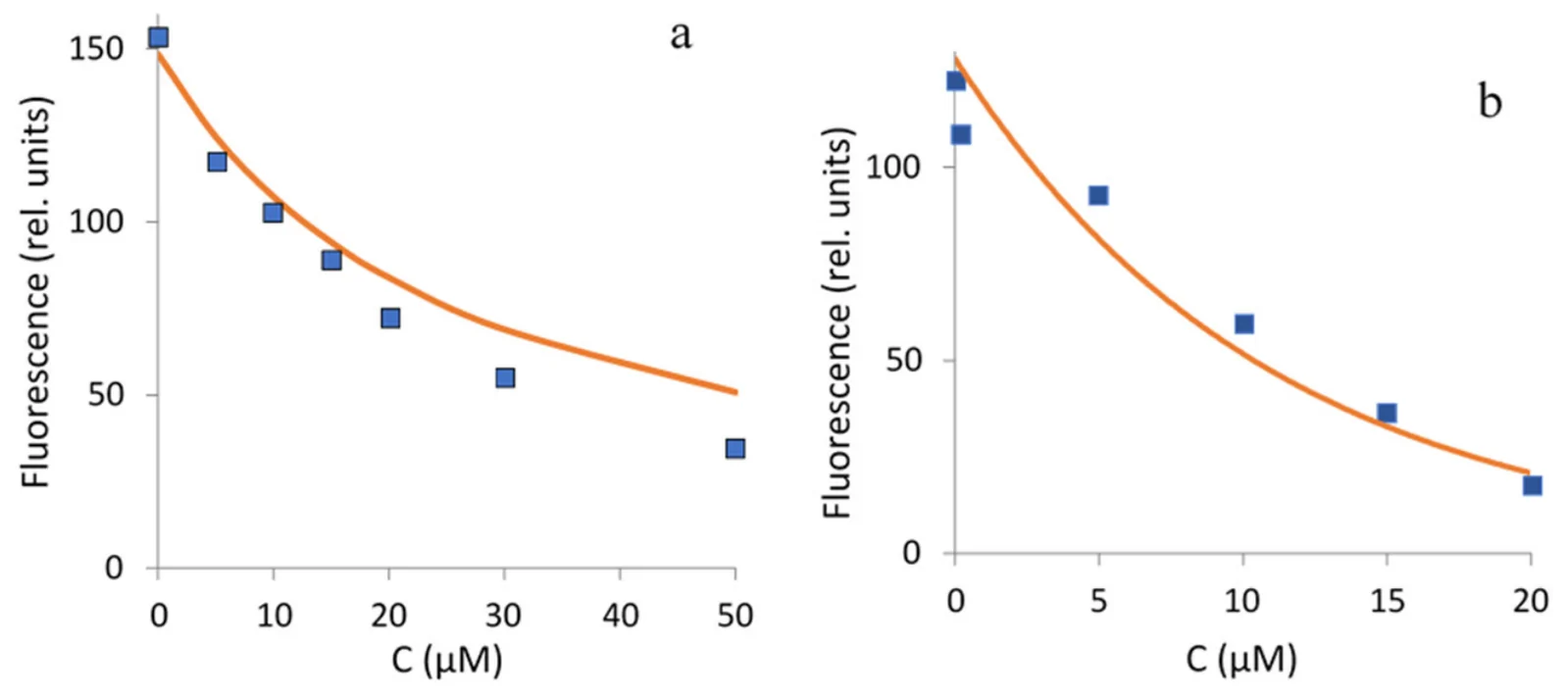
The experimental and theoretical dependences of the fluorescence intensity of dyes complexed with DNA (5 μM) with increasing concentration of **PdCo** in solution: (**a**) ethidium bromide in concentration of 5 μM; (**b**) Hoechst 33258 at a concentration of 1 μM.

To calculate the binding parameters, we used a model of the competitive binding of two ligands on a DNA template [40]. The results were processed according to the equation I = I_0_/(1 + C/K_d_), where I_0_ is the initial fluorescence intensity of a DNA/ethidium bromide solution or DNA/Hoechst 33258 solution; I represents the fluorescence intensity of a dye/DNA solution after the addition of **PdCo**; C is the concentration of **PdCo** added. Experimental data were approximated by theoretical curves (Figure 7). The **PdCo** binding constant was 26.1 ± 0.7 μM determined with ethidium bromide as a competitive dye and 7.6 ± 0.2 μM with Hoechst 33258, which indicates a preference for binding to the DNA groove over intercalation.

### 2.5. Molecular Docking

According to mass spectrometry data, the stable species is the {PhenPd(dmpz)_2_} fragment, which is most likely capable of binding to DNA. Consequently, we performed molecular docking for this fragment.

**Figure 8:**
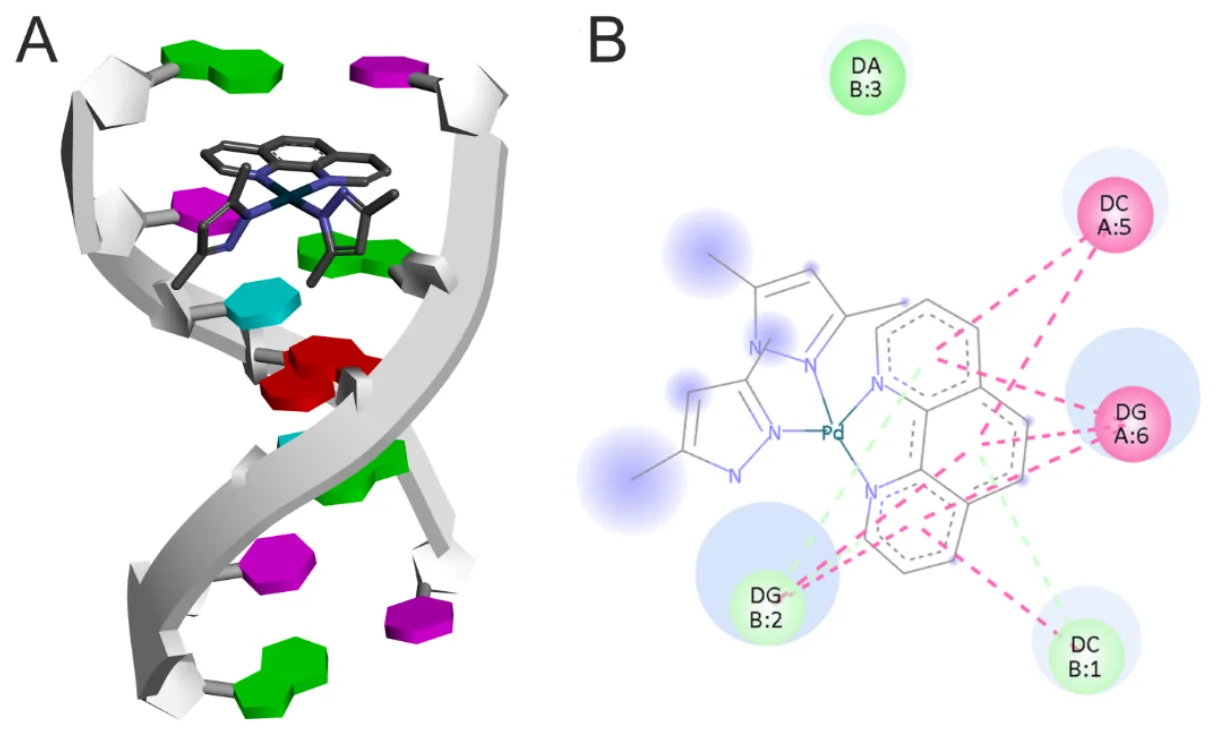
Molecular docking of intercalates of {PhenPd(dmpz)_2_} (**A**) in the human DNA bases (pdb id 1Z3F). Ligand interaction diagram (**B**) shows interactions with nucleotides of DNA at distances of 4 Å. The light purple atom background represents the solvent-accessible surface area of that atom.

{PhenPd(dmpz)_2_} contains a phenanthroline ligand, which allows it to intercalate between DNA nucleobases. Molecular docking shows that the planar aromatic structure of phenanthroline intercalates between adjacent base pairs, establishing stable π-π stacking interactions with the nucleic acid bases (Figure 8). {PhenPd(dmpz)_2_} aligns its major axis parallel to the Watson–Crick hydrogen bonds of the base pairs. The pyrazolate ligands are oriented within the major groove of DNA, significantly reducing their solvent accessibility. The binding affinity is −8.9 kcal/mol.

**Figure 9:**
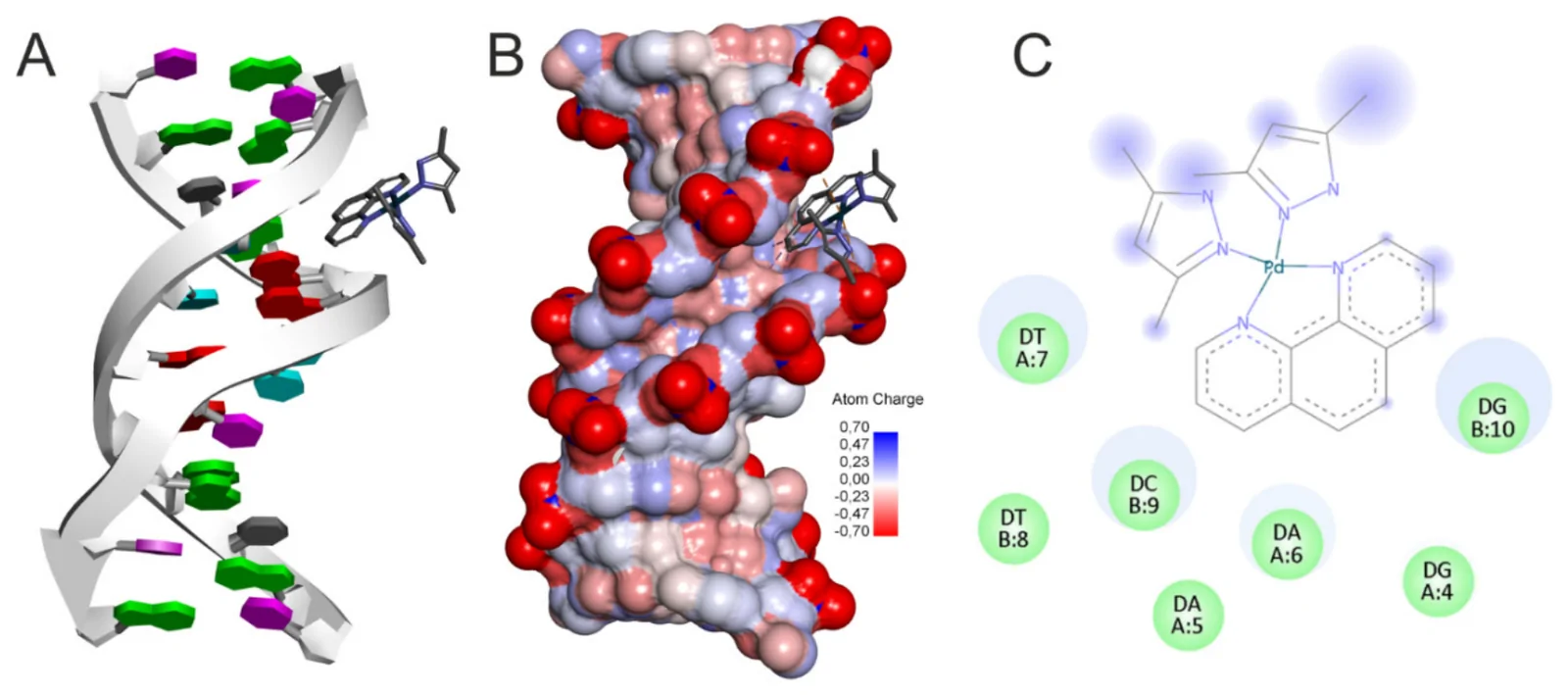
Molecular docking of the position of {PhenPd(dmpz)_2_} (**A**) in the human DNA minor groove (pdb id 4HLI). (**B**) DNA surface is colored according to the atom charge. Non-H-bond interactions are shown by the dotted lines. Ligand interaction diagram (**C**) shows interactions with nucleotides of DNA at distances of 4 Å. The light purple atom background represents the solvent-accessible surface area of that atom.

We also assessed the ability of {PhenPd(dmpz)_2_} binding in the major and minor grooves of DNA. It was found that, on the one hand, the compound is too small to effectively interact with elements of the major groove of DNA. On the other hand, the presence of two pyrazolate ligands sterically hinders the complete placement of {PhenPd(dmpz)_2_} in the minor groove (Figure 9). The phenanthroline ligand is located in the cavity of the DNA minor groove, forming π–alkyl interactions with the nucleobases (Figure 9B). Furthermore, {PhenPd(dmpz)_2_} is coordinated by palladium attractive charge interactions with the oxygen atoms of the phosphate groups that form the upper and lower edges of the minor groove. The binding affinity is −6.4 kcal/mol.

## 3. Materials and Methods

### 3.1. General Details

Commercial reagents and solvents were used for the synthesis: acetonitrile (≥99%), benzene, The starting complexes [Cu(Hdmpz)_2_(OOCMe)_2_], [Co_3_(dmpz)_4_(Hdmpz)_2_(OOCMe)_2_], were synthesized according to the methods [31,41]. IR spectra were recorded on a Perkin Elmer Spectrum 65 spectrophotometer (PerkinElmer Inc., Shelton, CT, USA) equipped with a Quest ATR Accessory (Specac) by the attenuated total reflectance (ATR) in the range of 400–4000 cm^–1^. The purity of the compounds was confirmed by elemental analysis. Microprobe analyses were carried out using a Carlo Erba EA 1108 Series CHNS Elemental Analyzer (Carlo Erba Instruments, Cornaredo, Italy). Positive ion electrospray mass spectra were obtained with a Series 1100 MSI detector HP spectrometer (Agilent, Palo Alto, CA, USA), using an acetonitrile mobile phase. Solutions for electrospray ionization mass spectrometry (ESI-MS) were prepared using reagent-grade methanol. The UV-vis spectra were obtained using a CF2000 spectrophotometer (OKB Spektr LLC, St. Petersburg, Russia) in 1% DMSO/H_2_O in the range of 220–400 nm.

### 3.2. Synthesis of [PdCo(phen)(µ-dmpz)_2_(OOCMe)_2_] (**PdCo**)

First, 0.1 mmol (0.087 g) [Co_3_(µ-dmpz)_4_(Hdmpz)_2_(OOCMe)_2_] was added to a solution containing 0.3 mmol (0.121 g) of [Pd(phen)(OOCMe)_2_] in 15 mL of acetonitrile and mixed at room temperature for 0.5 h. The resulting solution was concentrated to 5 mL and kept at a temperature of +5 °C for a day. Violet crystals in 60% yield (0.117 g) formed that were separated from the mother liquor by decantation. Anal. calc. C_26_H_30_CoN_6_O_5_Pd (taking into account the removed disordered water molecules): C 46.48; H 4.50, N 12.50. Found: C 46.68; H 4.21; N 12.10. FT-IR (ATR), ν/cm^−1^: 3429 w, 3053 w., 2918 w, 1623 s, 1576 s, 1521 s, 1415 s, 1389 s, 1332 m, 1315 s, 1223 m., 1145 w, 1110 w, 1044 s, 922 m, 847 s, 777 s, 716 s, and 669 m.

### 3.3. X-Ray Studies

The experimental data for the complex was obtained using Bruker APEX-II CCD four-circle diffractometer (Bruker AXS, Karlsruhe, Germany, Mo *K*α radiation, λ = 0.71073 Å) at 296 K in *ω*-scan mode, at the Center for Collective Use of the Kurnakov Institute RAS (Moscow, Russia). The raw data was treated with the APEX3 program suite [42]. Experimental intensities were corrected for absorption effects using SADABS [43]. The crystal structures were solved by direct methods [44] and refined by the full-matrix least-squares on *F*^2^ [45] using OLEX2 structural data visualization and analysis program suite [46]. All non-hydrogen atoms were refined with anisotropic thermal parameters. Two disordered water molecules were excluded from the final structure refinement using the SQUEEZE procedure.

Crystal Data and structure refinement for **PdCo** are presented in Table S2. CCDC № 2561973 entries contain the supplementary crystallographic data for the structure **PdCo**. These data were provided free of charge by The Cambridge Crystallographic Data Centre: http://www.ccdc.cam.ac.uk/structures (accessed on 25 September 2026).

Powder diffraction patterns were obtained using a Bruker D8 Advance diffractometer (Bruker AXS, Karlsruhe, Germany) equipped with a LynxEye detector with Bragg–Brentano geometry; the sample was finely dispersed on a zero-background silicon holder, λ(CuKα) = 1.54060 Å. The obtained data were processed using the TOPAS 4 software [47].

### 3.4. Cytotoxic Effect

The antiproliferative effects of the tested compounds were evaluated using the MTT colorimetric assay. A panel of human tumor cell lines was employed, including breast carcinoma (SKBR3, ATCC HTB-30), colorectal carcinoma (HCT116, ATCC CCL-247), and lung carcinoma lines (A549, ATCC CRM-CCL-185; NCI-H1299, ATCC CRL-5803). All cell lines were sourced from the American Type Culture Collection (ATCC) and verified by short-tandem repeat profiling, with confirmed absence of mycoplasma contamination. To assess the selectivity toward cancer cells, normal human dermal fibroblasts (HDFs, Lonza CC-2511) were used as a non-malignant control. Cultures were maintained in RPMI-1640 or DMEM (Gibco, Invitrogen) supplemented with 10% fetal bovine serum (Gibco, Invitrogen) and 1% penicillin/streptomycin at 37 °C under a 5% CO_2_ atmosphere. The selectivity index (SI) was determined as the ratio of IC_50_ values for non-cancerous cells to those for cancer cells.

For the assay, cells were plated into 96-well plates at a density of 3–5 × 10^3^ cells per well and allowed to adhere overnight at 37 °C, 95% humidity, and 5% CO_2_. Test compounds were dissolved in DMSO and serum-free medium to prepare a range of concentrations, which were then applied to the cells. The final DMSO content did not exceed 1.0% and was confirmed to be non-cytotoxic. Following 72 h of incubation with the compounds, 20 µL of MTT reagent (5 mg/mL) was added to each well, and the plates were further incubated at 37 °C. After 4 h, the medium was discarded, and 200 µL of DMSO was added per well to dissolve the purple formazan crystals. The optical density was measured at 540 nm using a Multiscan FC microplate reader (Thermo Fisher Scientific, Waltham, MA, USA).

Each experiment was performed in triplicate. IC_50_ values were derived from nonlinear regression analysis using GraphPad Prism 9.0 (Windows). The results are expressed as the mean ± standard deviation (SD).

### 3.5. Statistical Analysis

All experiments were carried out in triplicate. The IC_50_ values were calculated by a nonlinear regression curve using GraphPad Prism version 9.0 for Windows. The data of IC_50_ are presented as the mean ± standard deviation (SD). Statistical analysis of the cell cycle and apoptosis studies were performed using SPSS V. 22.0 by comparing the treatment groups to the control. Analysis of the data was performed using one-way analysis of variance (ANOVA) followed by Dunnett’s test. The data are expressed as the mean ± standard deviation (SD). In all cases, *p* < 0.05 was considered as statistically significant.

### 3.6. DNA Binding

The salmon sperm DNA was from PharmPack (Moscow, Russia); the ethidium bromide (EtBr) and Hoechst 33258 dyes were purchased from Sigma (Ronkonkoma, NY, USA).

CD spectra were recorded using the Jouan Mark III dichrograph (Jobin Yvon, Palaiseau, France) at 25 °C using quartz cuvettes with an optical path length of 1 cm. The DNA concentration was 50 μM. The 1 mM stock solution of **PdCo** was prepared in H_2_O.

Fluorescence spectra were recorded using a Cary Eclipse spectrofluorometer (Agilent, Santa Clara, CA, USA), employing a temperature-controlled cuvette maintained at 25 °C. The excitation wavelengths were 540 nm for EtBr and 360 nm for Hoechst 33258. In terms of fluorescence detection, emissions were monitored at 600 nm for EtBr and 480 nm for Hoechst 33258 during subsequent analyses. The measurements were carried out at fixed concentrations of both dyes and DNA, while changes in the fluorescence intensity were monitored to assess competitive binding interactions. The 0.1 mM stock solution of **PdCo** was prepared in H_2_O.

### 3.7. ROS Activity

The prooxidant properties of the palladium complexes were examined by a chemiluminescence-based approach utilizing the luminol oxidation reaction in the presence of hydrogen peroxide. Measurements were performed in phosphate buffer (100 mM, pH 7.4) at 37 °C. Chemiluminescence (CL) signals were detected in 2 mL plastic cuvettes with a 12-channel Lum-1200 chemiluminometer (DISoft, Moscow, Russia). The final concentrations used were 50 µM luminol (123072, Sigma-Aldrich, St. Louis, MO, USA) and 10 mM hydrogen peroxide (99.9%, Chimmed, Moscow, Russia). After registering the baseline signal for 60 s, an aqueous solution of the test complex was introduced (0.5–10 μM), bringing the total reaction volume to 1 mL. Integral CL intensity values were obtained by calculating the area under the kinetic curve over a 60-min period. Kinetic modeling of the luminol oxidation process was performed with Kinetic Analyzer 3.1 software (Moscow, Russia).

The superoxide dismutase (SOD)-mimicking activity of the palladium complexes was evaluated through a chemiluminescent assay based on lucigenin oxidation by superoxide anion radicals. These radicals were generated via xanthine oxidation catalyzed by xanthine oxidase in the presence of oxygen. Into a cuvette containing phosphate buffer (100 mM, pH 7.4), aliquots of xanthine (40 μM, #X0626, Sigma-Aldrich), lucigenin (40 μM, #393824, J&K, San Jose, CA, USA), and the complex (0.5–10 μM) were added. Following a 60-s background recording, xanthine oxidase (a = 11 mU/mL, #X1875–25UN, Sigma-Aldrich) was introduced. CL intensity was monitored at 37 °C using the Lum-1200 chemiluminometer.

### 3.8. Molecular Docking

Molecular docking was performed using AutoDock Vina 1.1.2 with default parameters [48]. The structure of pdb id 4HLI from which water molecules were removed was used as model of binding to human DNA. The structure of pdb id 1Z3F from which water molecules and ligands were removed was used as model of intercalation in DNA. The binding site was determined according to the position of the ellipticine model structure.

The structure of the dissociated palladium fragment was prepared in the BIOVIA Discovery Studio software package v24.1.0.23298 (BIOVIA, Dassault Systems, San Diego, CA, USA) from a 3D structure of the **PdCo** complex. Molecular docking was calculated to determine its position within the binding cavities of pdb 4HLI and 1Z3F, with a docking box of 24 × 24 × 30 Å and 26 × 18 × 16 Å, respectively. The interactions of the model ligand with nucleotide residues of DNA were calculated using BIOVIA Discovery Studio software package at a distance of 4 Å.

## 4. Conclusions

A novel heterometallic pyrazolate-bridged complex **PdCo**, with a structurally analogous **PdCu** complex, has been synthesized and fully characterized by X-ray diffraction, IR, UV spectroscopy, and mass spectrometry. The complexes **PdCo** and **PdCu** exhibit micromolar cytotoxicity against HCT116, A549, and SKBR3 cell lines; **PdCu** surpasses cisplatin by 2–2.5 times, with high selectivity, while PdCo demonstrates selectivity toward the SKBR3 line. The mechanism of action can include two complementary pathways: pro-oxidant activity, where **PdCu** exhibits peroxidase-like activity (20 times higher than that of **PdCo**), promoting oxidative stress; or DNA binding via the minor groove (K_d_ = 7.6 μM) with minor conformational changes to B-DNA (confirmed by molecular docking). The differences in cytotoxicity between the structurally similar **PdCo** and **PdCu** are determined by the nature of the partner metal: copper(II) in **PdCu**, due to its high redox activity (Cu^2+^/Cu^+^) and coordination sphere lability, generates ROS more efficiently, resulting in low IC_50_ values, whereas cobalt(II) in **PdCo** forms a more stable coordination site with lower redox activity, leading to higher IC_50_ values. Nevertheless, the reduced reactivity of the cobalt center accounts for the exceptional selectivity of **PdCo** toward the SKBR3 cell line.

## Figures and Tables

**Table 1:** Cytotoxicity of the compounds in human tumor cell lines and normal fibroblasts after 72 h.

Code	IC_50_, µM				SI		
	HCT116	A549	SKBR3	HDF	HCT116	A549	SKBR3
**PdCo**	46 ± 4	67 ± 8	18 ± 1	137 ± 14	2.9	2.0	7.7
**PdCu**	3 ± 0.3 *	2 ± 0.6 *	8 ± 2 *	16 ± 2	5.4	7.0	2.0
[Pd(phen)(OAc)_2_]	11 ± 1	8.6 ± 0.5	12 ± 1	26 ± 3	2.5	3.3	2.2
[Co_3_(dmpz)_4_(Hdmpz)_2_(OAc)_2_]	>100	>100	48 ± 8	>100	-	-	>2
Cp ^a^	8 ± 0.5	5 ± 0.3	8 ± 0.6	23 ± 2	3.0	5.0	2.9

^a^ Data was obtained in our previous study [32]. * *p* < 0.05 vs. cisplatin.

## Data Availability

The original contributions presented in this study are included in the article/Supplementary Material. Further inquiries can be directed to the corresponding author.

## Supplementary Materials

The following supporting information can be downloaded at https://www.mdpi.com/article/10.3390/ijms27209009/s1.

## Abbreviations

The following abbreviations are used in this manuscript:

FTIR	Fourier Transform Infrared Spectroscopy
DNA	Deoxyribonucleic acid
UV	Ultraviolet
ROS	Reactive oxygen species
Hdmpz	3,5-dimethylpyrazole
DMSO	Dimethylsuldoxide
