# Supplementary material for: Pyrazolate-Bridged PdCo and PdCu Complexes as Dual DNA-Binding and ROS-Driven Cytotoxic Agents
Source: Int J Mol Sci. 2026 Oct 11;27(20):9009. doi: 10.3390/ijms27209009 (PMC13704585; doi:10.3390/ijms27209009)

checkCIF/PLATON report

Structure factors have been supplied for datablock(s) 1

THIS REPORT IS FOR GUIDANCE ONLY. IF USED AS PART OF A REVIEW PROCEDURE FOR PUBLICATION, IT SHOULD NOT REPLACE THE EXPERTISE OF AN EXPERIENCED CRYSTALLOGRAPHIC REFEREE.

No syntax errors found. CIF dictionary Interpreting this report

Datablock: 1

Bond precision:	C-C = 0.0070 A		Wavelength=0.71073
Cell:	a=9.9625(6)	b=17.3145(11)	c=17.6394(11)
	alpha=88.608(1)	beta=81.659(1)	gamma=79.313(1)
Temperature:	296 K		

	Calculated	Reported
Volume	2958.3(3)	2958.3(3)
Space group	P -1	P -1
Hall group	-P 1	-P 1
Moiety formula	C26 H28 Co N6 O4 Pd [+ solvent]	2(C26 H28 Co N6 O4 Pd)
Sum formula	C26 H28 Co N6 O4 Pd [+ solvent]	C52 H56 Co2 N12 O8 Pd2
Mr	653.87	1307.74
Dx,g cm-3	1.468	1.468
Z	4	2
Mu (mm-1)	1.208	1.208
F000	1324.0	1324.0
F000’	1321.82	
h,k,lmax	12,21,21	12,21,21
Nref	11640	11377
Tmin,Tmax	0.886,0.886	0.715,0.826
Tmin’	0.886	

```
Correction method= # Reported T Limits: Tmin=0.715 Tmax=0.826
AbsCorr = MULTI-SCAN

Data completeness= 0.977          Theta(max)= 25.998

                                                    wR2(reflections)=
R(reflections)= 0.0421( 7728)                       0.0971( 11377)

S = 0.968                  Npar= 701
```

The following ALERTS were generated. Each ALERT has the format
test-name_ALERT_alert-type_alert-level.
Click on the hyperlinks for more details of the test.

Alert level C

```
ABSTY02_ALERT_1_C  An _exptl_absorpt_correction_type has been given without
            a literature citation. This should be contained in the
            _exptl_absorpt_process_details field.
            Absorption correction given as multi-scan
PLAT241_ALERT_2_C High  MainResAtom  Ueq as Compared to Neighbours        O7 Check
PLAT242_ALERT_2_C Low   MainResAtom  Ueq as Compared to Neighbours       C50 Check
PLAT242_ALERT_2_C Low   MainResAtom  Ueq as Compared to Neighbours       C63 Check
PLAT242_ALERT_2_C Low   MainResAtom  Ueq as Compared to Neighbours       C39 Check
PLAT242_ALERT_2_C Low   MainResAtom  Ueq as Compared to Neighbours       C61 Check
PLAT790_ALERT_4_C Centre of Gravity not Within Unit-Cell: Resd.  #         1 Note
              C26 H28 Co N6 O4 Pd
PLAT905_ALERT_3_C Negative K value in the Analysis of Variance ...     -1.615 Report
PLAT910_ALERT_3_C Missing FCF Reflection(s) Below Theta(Min)[Deg]=       2.37 Note
                1  0  0,   0  1  0,   1  1  0,   0 -1  1,   0  0  1,   1  0  1,
                0  1  1,   0  0  2,
PLAT911_ALERT_3_C Missing FCF Refl Between Thmin & STh/L=    0.600        197 Report
               -2  1  0,   0  2  0,   0  3  0,   0  4  0,  -1  5  0,   0  5  0,
                0 -8  1,   0 -5  1,   0 -3  1,   0 -2  1,   0  2  1,   1  2  1,
                1  3  1,   0  4  1,   1  4  1,   1  5  1, -11 -7  2, -11 -6  2,
                0 -5  2,  -1 -3  2,   0 -3  2,   1 -2  2,  -1 -1  2,   1 -1  2,
                0  1  2,   0  2  2,  -1  5  2,   2  5  2, -11 -8  3, -11 -7  3,
                   (  167 More NOT listed: see .ckf listing file)
```

Alert level G

```
PLAT003_ALERT_2_G Number of Uiso or U(i,j)  Restrained non-H-Atoms         2 Report
PLAT041_ALERT_1_G Calc. and Reported SumFormula    Strings  Differ     Please Check
              Calc: C26 H28 Co N6 O4 Pd
              Rep.: C52 H56 Co2 N12 O8 Pd2
PLAT042_ALERT_1_G Calc. and Reported MoietyFormula Strings  Differ     Please Check
              Calc: C26 H28 Co N6 O4 Pd
              Rep.: 2(C26 H28 Co N6 O4 Pd)
PLAT045_ALERT_1_G Calculated and Reported Z Differ by a Factor ...         2 Check
PLAT154_ALERT_1_G The s.u.'s on the Cell Angles are Equal ..(Note)     0.001 Degree
PLAT171_ALERT_4_G The CIF-Embedded .res File Contains EADP Records         1 Report
PLAT186_ALERT_4_G The CIF-Embedded .res File Contains ISOR Records         1 Report
PLAT301_ALERT_3_G Main Residue  Disorder .............(Resd    1)        3% Note
PLAT606_ALERT_4_G Solvent Accessible VOID(S) in  Crystal Structure         ! Info
```

```
PLAT790_ALERT_4_G Centre of Gravity not Within Unit-Cell: Resd.  #          2 Note
              C26 H28 Co N6 O4 Pd
PLAT794_ALERT_5_G Tentative Bond Valency for Pd1       (II)      .       2.32 Info
PLAT794_ALERT_5_G Tentative Bond Valency for Pd2       (II)      .       2.33 Info
PLAT794_ALERT_5_G Tentative Bond Valency for Co1       (II)      .       1.78 Info
PLAT860_ALERT_3_G Number of Least-Squares Restraints ............          12 Note
PLAT868_ALERT_4_G ALERTS Due to the Use of _smtbx_masks Suppressed          ! Info
PLAT883_ALERT_1_G Absent Datum for _atom_sites_solution_primary ..     Please Do !
PLAT912_ALERT_4_G Missing # of FCF Reflections Above STh/L=  0.600         59 Note
PLAT933_ALERT_2_G Number of HKL-OMIT Records in Embedded .res File         37 Note
               -2  1  0,  -1 -3  2,  -1 -1  2,  -1  0  3,  -1  1  3,  -1  1  4,
               -1  5  0,  -1  5  2,   0 -8  1,   0 -5  1,   0 -5  2,   0 -3  1,
                0 -3  2,   0 -2  1,   0 -2  3,   0 -1  1,   0  0  1,   0  1  0,
                0  1  1,   0  1  2,   0  2  0,   0  2  1,   0  2  2,   0  3  0,
                0  4  0,   0  4  1,   0  5  0,   1 -2  2,   1 -1  2,   1  2  1,
                1  3  1,   1  4  1,   1  5  1,   2 -1  3,   2  4  3,   2  5  2,
                4  1  7,
PLAT941_ALERT_3_G Average HKL Measurement Multiplicity ..........         2.0 Low
PLAT967_ALERT_5_G Note: Two-Theta Cutoff Value in Embedded .res ..       52.0 Degree
PLAT969_ALERT_5_G The 'Henn et al.' R-Factor-gap value ..........       1.775 Note
              Predicted wR2: Based on SigI**2  5.47 or SHELX Weight 10.04 Note
PLAT978_ALERT_2_G Number C-C Bonds with Positive Residual Density.          1 Info
PLAT992_ALERT_5_G Repd & Actual _reflns_number_gt Values Differ by         -2 Check
```

```
   0 ALERT level A = Most likely a serious problem - resolve or explain
   0 ALERT level B = A potentially serious problem, consider carefully
  10 ALERT level C = Check. Ensure it is not caused by an omission or oversight
  23 ALERT level G = General information/check it is not something unexpected

   6 ALERT type 1 CIF construction/syntax error, inconsistent or missing data
   8 ALERT type 2 Indicator that the structure model may be wrong or deficient
   6 ALERT type 3 Indicator that the structure quality may be low
   7 ALERT type 4 Improvement, methodology, query or suggestion
   6 ALERT type 5 Informative message, check
```

It is advisable to attempt to resolve as many as possible of the alerts in all categories. Often the minor alerts point to easily fixed oversights, errors and omissions in your CIF or refinement strategy, so attention to these fine details can be worthwhile. It is up to the individual to critically assess their own results and, if necessary, seek expert advice.

PLATON version of 23/04/2026; check.def file version of 30/03/2026

---

duplicate check

No duplication found

Datablock 1 - ellipsoid plot

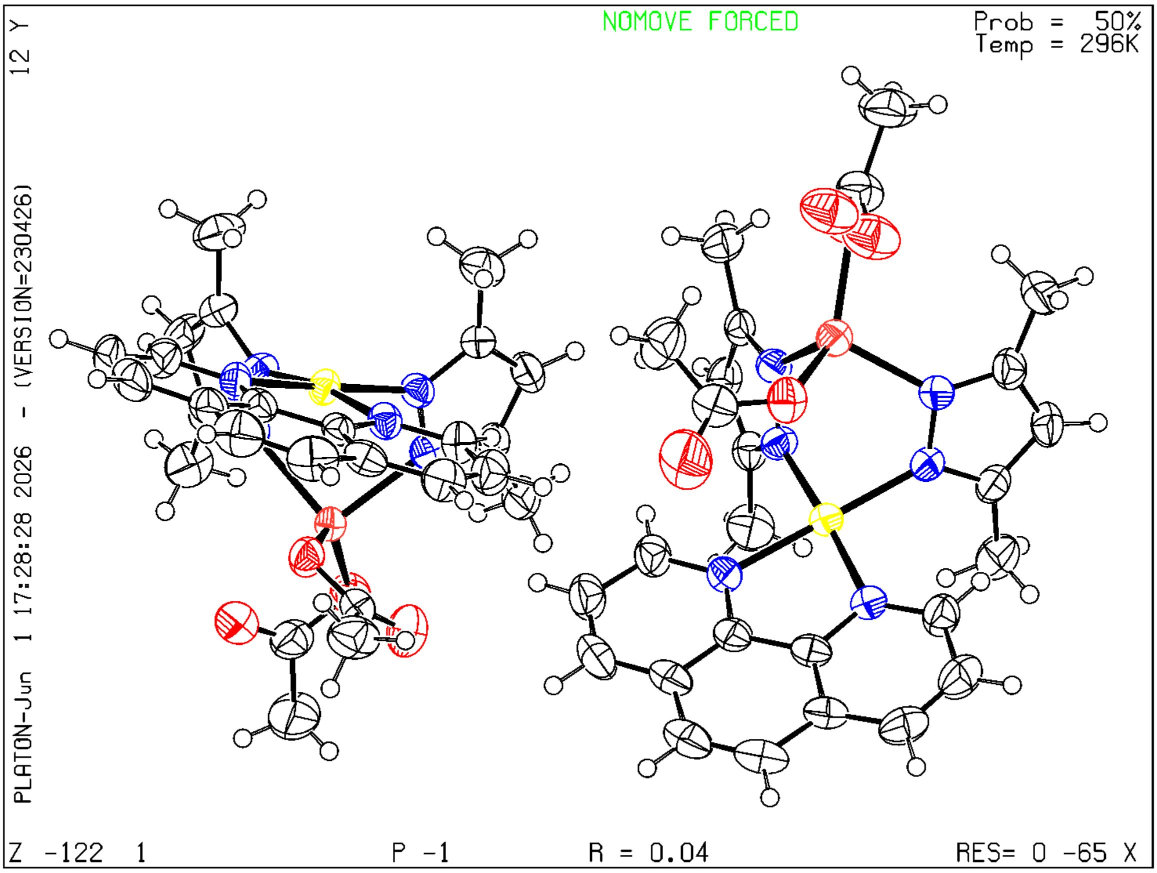

Supplement: Supplementary file 1 [file ijms-27-09009-s001.zip › checkcif (10).pdf]
