# Supplementary material for: Pyrazolate-Bridged PdCo and PdCu Complexes as Dual DNA-Binding and ROS-Driven Cytotoxic Agents
Source: Int J Mol Sci. 2026 Oct 11;27(20):9009. doi: 10.3390/ijms27209009 (PMC13704585; doi:10.3390/ijms27209009)
Supplement: Supplementary file 1 [file ijms-27-09009-s001.zip › Suppl.pdf]

Supporting Information

Pyrazolate-Bridged PdCo and PdCu Complexes as Dual DNA-Binding and ROS-Driven Cytotoxic Agents

Marina A. Uvarova[a*], Arina D. Filippova[a], Maxim A. Shmelev[a], VitaliaV. Kulikova[b], Yaroslav V. Kozmenko[b], Svetlana V. Revtovich[b], Darina V. Sokolova[c], Vadim S. Pokrovsky[c,d], Alexandra P. Kaverina[b], Pavel N. Solyev[b], Igor L. Eremenko[a]

[a] *N.S. Kurnakov Institute of General and Inorganic Chemistry of the Russian Academy of Sciences, Leninsky Prosp. 31, 119991 GSP-1, Moscow, Russian Federation*

Fax (495) 952 1279. E-mail: yak_marin@mail.ru

[b] *Engelhardt Institute of Molecular Biology, Russian Academy of Sciences, Vavilova str., 32, 119991, Moscow, Russian Federation*

[c] *N.N. Blokhin National Medical Research Center of Oncology, Kashirskoe Shosse 23, 115522, Moscow, Russian Federation*

[d] *RUDN University, Miklukho-Maklaya 6, 117198, Moscow, Russian Federation*

Table S1. Selected interatomic distances (Å) and angles (deg) in **PdCo**.

Bond	Length	angle	deg
Pd(1) –N(3)	1.992(4)	N(1)Pd(1)N(6)	93.88(18)
Pd(1)–N(6)	2.035(5)	N(6)Pd(1)N(5)	81.54(18)
Pd(1) – N(1)	1.988(4)	N(1)Pd(1)N(3)	87.71(18)
Pd(1) – N(5)	2.040(4)	N(1)Pd(1)N(5)	97.21(19)
Co(1) –O(3)	1.999(4)	N(9)Pd(2)N(7)	87.68(18)
Co(1) – N(4)	2.061(4)	N(9)Pd(2)N(11)	95.24(19)
Co(1) – N(2)	2.063(4)	N(7)Pd(2)N(12)	95.80(18)
Co(1) – O(2)	2.012(4)	N(11)Pd(2)N(12)	81.36(19)
Pd(2) –N(9)	1.977(4)	O(3)Co(1)N(4)	105.06(17)
Pd(2)–N(12)	2.039(4)	O(3)Co(1)N(2)	102.57(17)
Pd(2) – N(7)	1.989(4)	N(4)Co(1)N(2)	93.95(17)
Pd(2) – N(11)	2.025(4)	O(2)Co(1)N(4)	106.40(18)
Co(2) –O(5)	1.954(4)	O(5)Co(2)N(10)	104.83(18)
Co(2) – N(10)	2.021(4)	O(5)Co(2)N(8)	105.29(18)
Co(2) – N(8)	2.033(5)	N(10)Co(2)N(8)	98.95(19)
Co(2) – O(7)	1.947(4)	O(7)Co(2)N(10)	107.7(2)
Pd….Co	3.215(3)		

Table S2. Crystal data and structure refinement for **PdCo**.

Parameter	Value
Empirical formula	$C_{52}H_{56}Co_2N_{12}O_8Pd_2$
Formula weight (M)	1307.74 g/mol
Crystal system	Triclinic
Space group	P-1 (no. 2)
a	9.9625(6) Å
b	17.3145(11) Å
c	17.6394(11) Å
α	88.6080(10)°
β	81.6590(10)°
γ	79.3130(10)°
Volume (V)	2958.3(3) Å^3
Z	2
Temperature (T)	296.15 K
μ(MoKα)	1.208 mm^{-1}
*D*_calc	1.468 g/cm^3
Reflections measured	23158 (4.73° ≤ 2Θ ≤ 51.996°)
Unique reflections	11377 (*R_int* = 0.0420, *R_sigma* = 0.0689)
R_1 (I > 2σ(I))	0.0421
wR_2 (all data)	0.0971

Table S3. Table of π···π interactions in crystal packing of complex **PdCo**.

Interactions	Cg···Cg, Å	Cg···Perp, Å	Symmetry code	α, deg
phen···phen	3.836(3)	3.389(2)	2-x, 2-y, 1-z	3.5(2)
phen···phen	3.919(3)	3.374(2)	4-x, 2-y, -z	0.0(2)

Table S4. Hydrogen bonds in the crystal packing of complex **PdCo**.

Interactions	Distance, Å				D – H···A, °
	D–H	Symmetry code	H···A	D···A	
C(55)–H(55)···O(6)	0.93	1-x,1-y,1-z	2.22	3.121(6)	164
C(62)–H(62)···O(3)	0.93	1+x,y,z	2.42	3.186(6)	139
C(81)–H(81)···O(4B)	0.93		2.39	3.177(6)	142

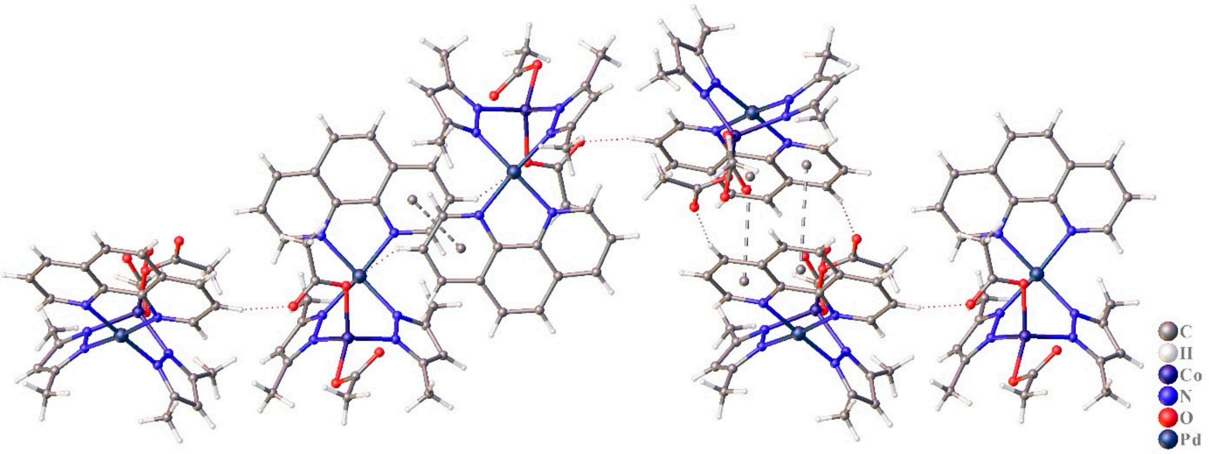

Figure S1. Fragment of the crystal packing of compound **PdCo**. Dashed lines indicate hydrogen bonds and π···π interactions.

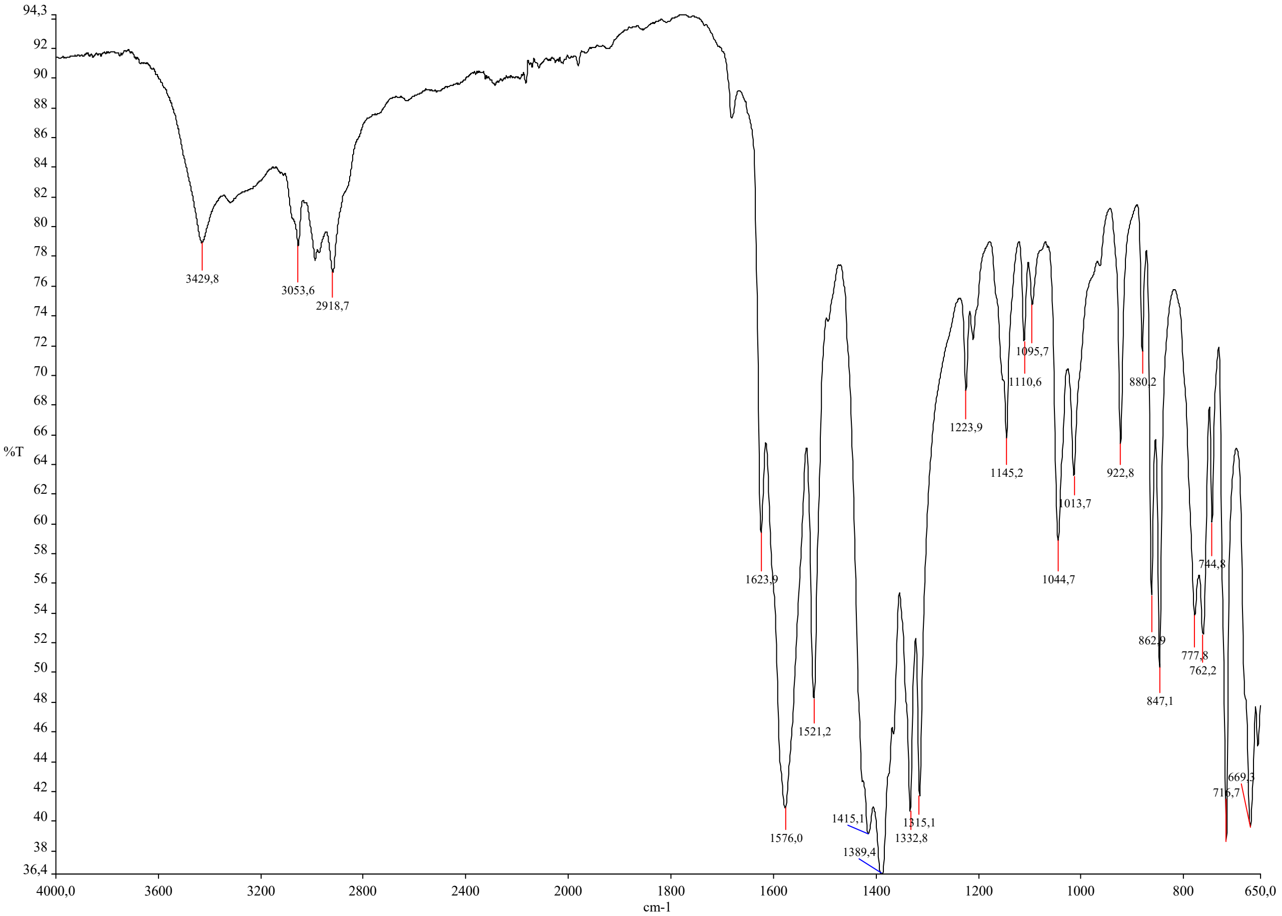

Figure S2. FT IR of **PdCo**

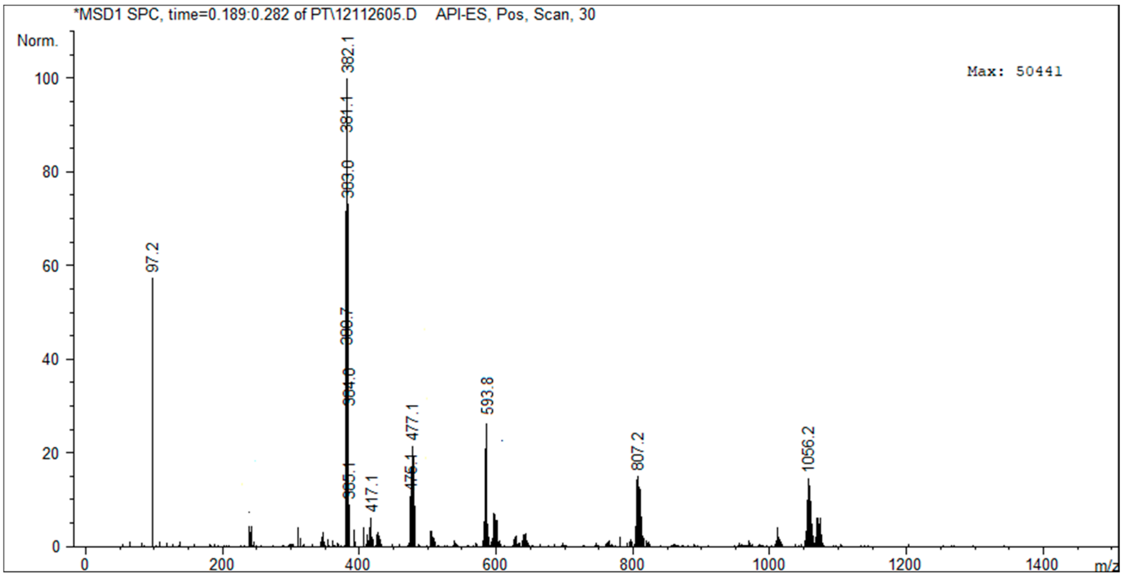

Figure S3. ESI Mass spectrum of PdCo

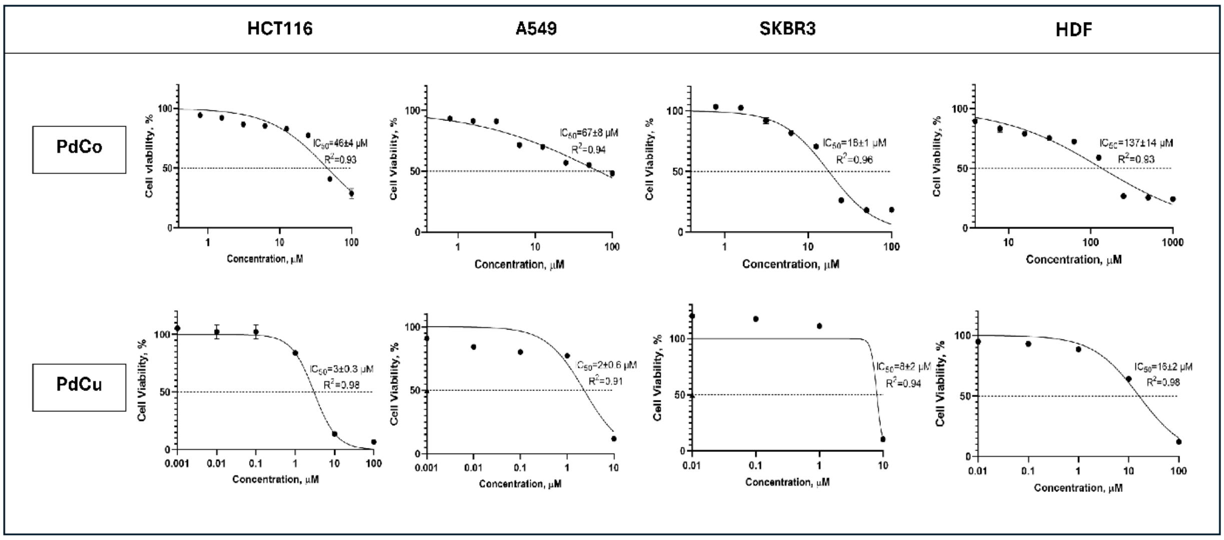

Figure S4. Survival of HCT116, A549 and SKBR3 cells and non-cancerous cells (HDF) incubated with **PdCo** and **PdCu** complexes. Co-incubation time 72 h

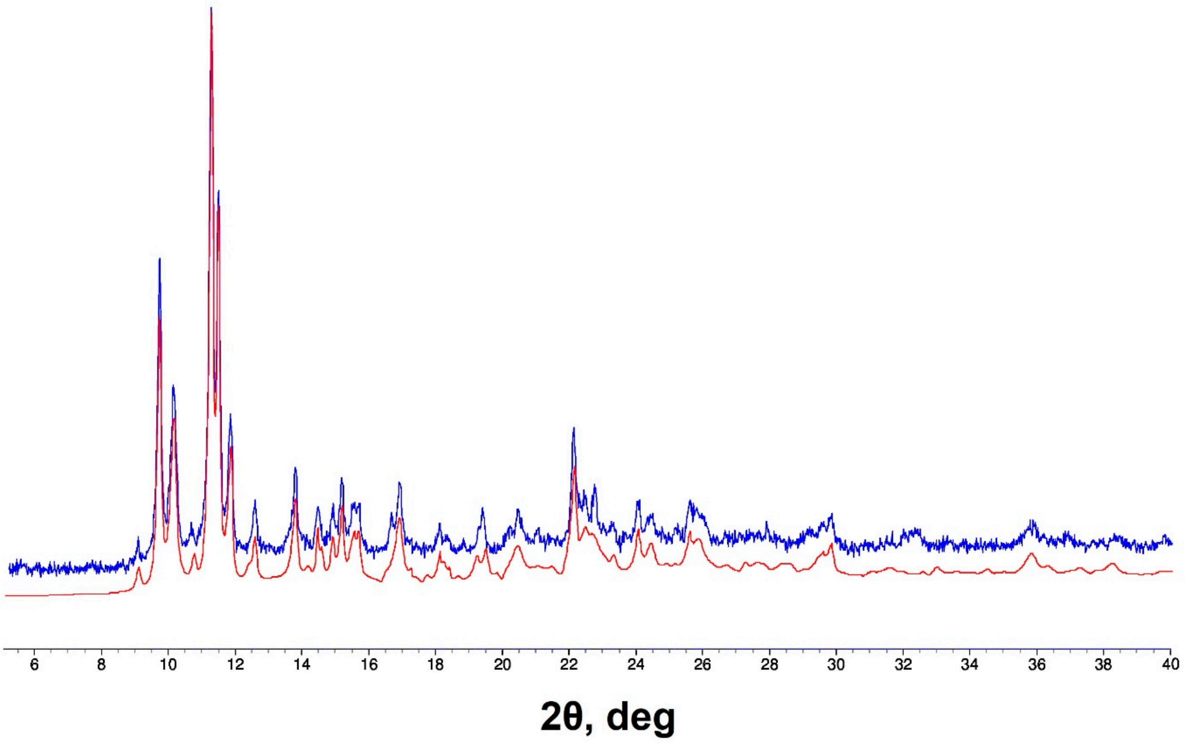

Figure S5. PXRD patterns of **PdCo** by the simulated from crystal data (red) and experimental (blue)

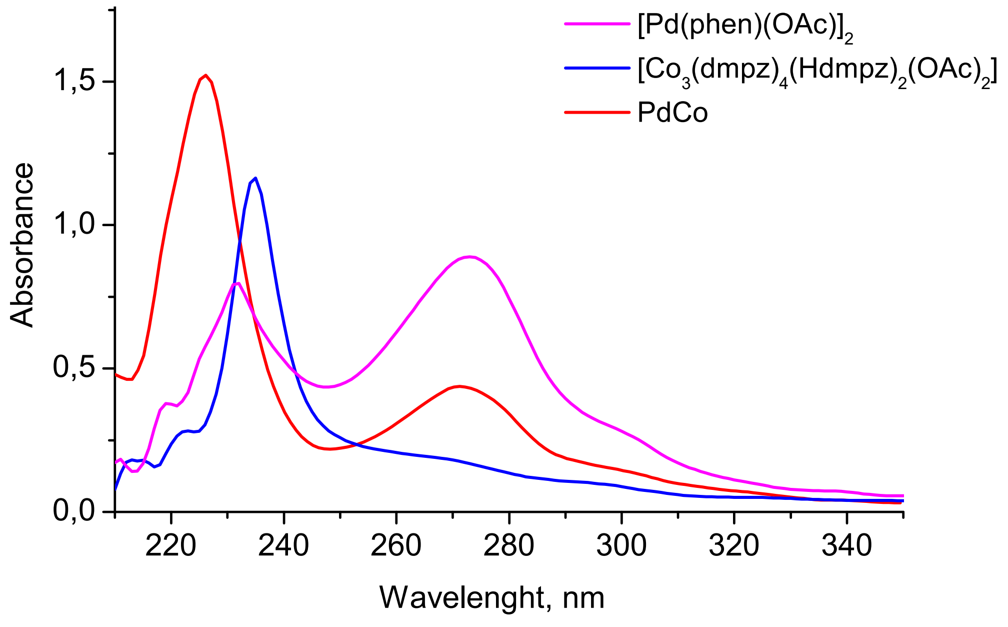

Figure S6. UV-vis spectra of **PdCo** and starting compounds [Pd(phen)$(OAc)_2$] and [Co_3(μ-dmpz)$_4$(Hdmpz)$_2$(OAc)$_2$] (C=10^{-5} M) in 1% DMSO/H_2O solution at room temperature.

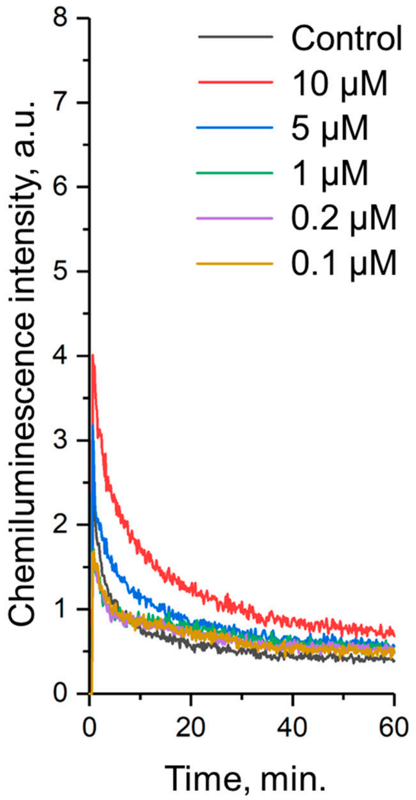

Figure S7. Experimental kinetic curves of chemiluminescence of luminol in the presence of hydrogen peroxide and mononuclear [Pd(phen)(OAc)$_2$]. The measurements were carried out in a phosphate buffer solution at 37°C.
